# Retraction: Mesenteric Panniculitis and COVID-19: A Rare Association

**DOI:** 10.7759/cureus.r128

**Published:** 2024-01-25

**Authors:** Israa A Alyousef, Zainab A Alsaileek, Manar A Alabdulsalam, Muhannad A Almohanna, Naif A Alshaqhaa, Mohammed M Alqahtani, Anas A Al Alyany, Muhannad A Alzahrani, Hawazin A Fallatah, Jalal I Alqadhib, Abdulaziz M Alhawsawi, Abdulelah D Alsuhaymi, Ali M Alasmari, Abdullah J Alshareef, Faisal Al-Hawaj

**Affiliations:** 1 College of Medicine, Dar Al Uloom University, Riyadh, SAU; 2 College of Medicine, Qassim University, Buraydah, SAU; 3 College of Medicine, Almaarefa University, Riyadh, SAU; 4 College of Medicine, Jordan University of Science and Technology, Irbid, JOR; 5 College of Medicine, Al-Baha University, Al-Baha, SAU; 6 College of Medicine, King Abdulaziz University, Jeddah, SAU; 7 College of Dentistry, Imam Abdulrahman Bin Faisal University, Dammam, SAU; 8 General Practice, Madinah General Hospital, Medina, SAU; 9 College of Medicine, Hunan Normal University, Changsha, CHN; 10 College of Medicine, Umm Al-Qura University, Mecca, SAU; 11 College of Medicine, Taif University, Taif, SAU; 12 College of Medicine, Imam Abdulrahman Bin Faisal University, Dammam, SAU

The Editors-in-Chief have retracted this article. Concerns were raised regarding the identity of the authors on this article. Specifically, Faisal Alhaway and Malak Shammari have stated that they were added to this article without their knowledge or approval. The identity of the other authors could also not be verified. As the appropriate authorship of this work cannot be established, the Editors-in-Chief no longer have confidence in the results and conclusions of this article.

